# Clinical and Molecular Analysis of Four Patients With 11β-Hydroxylase Deficiency

**DOI:** 10.3389/fped.2020.00410

**Published:** 2020-07-24

**Authors:** Qiaoli Zhou, Dandan Wang, Chunli Wang, Bixia Zheng, Qianqi Liu, Ziyang Zhu, Zhanjun Jia, Wei Gu

**Affiliations:** ^1^Department of Endocrinology, Children's Hospital of Nanjing Medical University, Nanjing, China; ^2^Nanjing Key Laboratory of Pediatrics, Children's Hospital of Nanjing Medical University, Nanjing, China

**Keywords:** *CYP11B1* gene, 11β-hydroxylase deficiency, virilization, final height impairment, hypertension

## Abstract

**Objective:** 11β-hydroxylase deficiency (11βOHD) is a rare autosomal recessive disorder caused by mutations in the *CYP11B1* gene. It is characterized by virilization, hypertension, and significant final height impairment. In this study, we aim to investigate the clinical and molecular characteristics of four unrelated Chinese patients with 11βOHD disorder.

**Methods:** The clinical information of four 11βOHD patients were carefully reviewed. Genetic analysis was performed using next-generation sequencing (NGS) based panel analysis. NGS coverage depth was analyzed to detect exonic copy-number variants (CNVs) on patient 1. Quantitative PCR (qPCR) was subsequently performed to confirm the CNVs detected from the NGS coverage depth analysis.

**Results:** The mean age of the patients at diagnosis was 4.7 years (range, 2.0–9.3 years). Two genetically female patients (patients 1 and 2) with 11βOHD presented severe virilization of external genitalia and were raised as males. Two genetically male patients (patients 3 and 4) presented precocious puberty. Additionally, patients 1, 3, and 4 presented with hypertension. In patient 4, unilateral adrenal mass was detected and removed at the age of 9 years. Interestingly, the height of patient 4 (174.4 cm, +6.7 SD) wasn't impaired and reached his mid-parental height (173 cm). Three novel variants in the *CYP11B1* gene (c.1150_1153del, c.217C>T, and c.400G>C) were identified by NGS. Various bioinformatics tools revealed potential pathogenic effects for the novel variants, and evolutionary-conservation revealed that the novel missense variant affected an amino acid that is highly conserved among species. Furthermore, NGS coverage depth analysis and qPCR identified a novel heterozygous deletion of exons 1–6 in patient 1.

**Conclusion:** Our study expands the spectrum of mutations of the *CYP11B1* gene in Chinese population. In addition, We reported the first case of a patient with classical 11βOHD disorder, whose final height wasn't compromised.

## Introduction

Congenital adrenal hyperplasia (CAH) is a group of autosomal recessive disorders caused by defects in various enzymes that are involved in the synthesis of cortisol from cholesterol by the adrenal cortex (1). The most common form of CAH is 21-hydroxylase deficiency (21OHD), accounting for 90–95% of all CAH cases (2). In contrast, CAH due to the deficiency of 11β-hydroxylase (11βOHD) accounts for ~5–8% of all cases and has an annual incidence of 1/100,000–200,000 live births (3). Female newborns can be identified with ambiguous genitalia as in 21OHD. However, boys are usually not diagnosed by newborn screening of 17-hydroxyprogesterone (17OHP), which is not as high in 11βOHD as it is in 21OHD (4, 5).

11βOHD is caused by mutations in the *CYP11B1* gene which is located on chromosome 8q21, ~40 kb from the highly identical *CYP11B2* gene (6). The *CYP11B1* gene includes 9 exons and encodes a 503 amino acid protein which belongs to the cytochrome P450 system. Variants that lead to deactivated CYP11B1 can abolish or reduce the activity of the 11β-hydroxylase which converts 11-deoxycortisol to cortisol and 11-deoxycorticosterone (DOC) to corticosterone in the mitochondria of the adrenal cortex. Reduced activity of 11β-hydroxylase leads to decreased cortisol secretion, accumulation of glucocorticoid and mineralocorticoid precursors, and excessive adrenal androgen biosynthesis. The classical and more severe form of 11βOHD is most likely caused by severe impairment of 11β-hydroxylase activity (residual activity of <10%) (7). Hypertension due to accumulation of the potent mineralocorticoid DOC, occurs in approximately two-third of the cases, often early in life (8, 9). In affected females, the excess androgens result in varying degrees of enlargement of the clitoris, fusion of the labioscrotal folds, and the vaginal and urogenital confluence. Both girls and boys with classical 11βOHD undergo rapid postnatal growth with sexual precocity and skeletal maturation, leading to short stature in adulthood (10, 11). In this study, we performed a genetic and phenotypic analysis of four Chinese patients with 11βOHD, including two 46,XX females misassigned to the male gender at birth and reared as males, and two 46,XY males.

## Materials and Methods

### Subjects

Four unrelated Chinese patients were investigated in this study. Patients 1 and 2 were genetically females but raised as males due to severe virilization before the diagnosis. The clinical diagnosis of the patients was based on evaluation of clinical symptoms and laboratory findings.

### Next-Generation Sequencing (NGS) Based Panel Analysis

Informed consent was obtained from all patients, peripheral blood samples were then collected from the patients and their parents. Genomic DNA was isolated from peripheral leukocytes using a DNA isolation kit (Tiangen, China) according to the manufacturer's instructions. Molecular studies were performed using GenCap congenital adrenal disorders capture kit (MyGenostics GenCap Enrichment technologies, China). The DNA probes were designed to tile along the exon regions of the 44 genes associated with congenital adrenal disorders (*CYP11B1, CYP11B2, DAX1, SF-1, PRKCAC, DHCR7, GK2, PDE8B, LHX4, ARMC5, MC2R, GK, H6PD, ABCD1, SOX3, GNAS, MRAP, POMC, HSD11B1, MKS1, CYP21A2, NNT, TBX19, MEN1, MCM4, REN, AIRE, CYP17A1, NR3C1, PCSK1, TXNRD2, CYP11A1, RXRA, PRKAR1A, HESX1, HSD3B2, GLCCI1, TP53, POR, RXRB, PDE11A, PROP1*, and *STAR*). Sanger sequencing was performed to confirm the identified variants. Additionally, NGS coverage depth was analyzed to detect exonic copy-number variants (CNVs) using an in-house algorithm (12).

### DNA Quantitative PCR (qPCR)

Using the primer pair sequences listed in Supplementary Table 1. qPCR was performed using the AceQ qPCR SYBR Green Mix (Vazyme Biotech Co., Ltd). The copy number of the *CYP11B1* gene was measured by subtracting the Ct values of the four exons (Exons 2, 5, 6, and 8) from an endogenous control (GAPDH) gene by using the 2-ΔΔCt method.

### Bioinformatics Analysis

Three software: PolyPhen-2 (http://genetics.bwh.harvard.edu/pph2/), Provean (http://provean.jcvi.org/), and SIFT (http://sift.jcvi.org/) were performed to predict the damaging effect of the identified mutations on CYP11B1 function. The amino acid sequences of human CYP11B1 protein were aligned with those in the homologous proteins from different species. The putative effect of the novel missense mutation was analyzed using the X-ray structure of the human CYP11B1 as a template (PDB accession number: 6M7X).

## Results

### Clinical Characteristics and Serum Hormone Levels

Clinical features and laboratory evaluations of the patients are listed in Tables 1, 2. All patients were born to non-consanguineous parents at full-term gestation. Patients 1 and 2 were found to have a (46,XX) karyotype while patients 3 and 4 had a karyotype of (46,XY).

**Table 1:** Clinical and genetic findings of four patients with 11βOHD.

**Case No**.	**Age**	**Sex of rearing**	**Karyotype**	**Height cm**	**Weight kg**	**BA y**	**Blood pressure mmHg (systolic/diastolic)**	**External genitalia Prader, Penis cm, Testis ml**	**Therapy**	***CYP11B1* variants**
P1	2.8	Male	46,XX	112.6 (+4.4 SD)	22.5 (+4.3 SD)	10	120/80 (>99th/>99th)	5, 4,–	hydrocortisone Spironolactone	p.(Arg384Trpfs*45) Exons 1-6del
P2	2	Male	46,XX	97.4 (+2.8 SD)	14.5 (+1.8 SD)	5.5	99/60 (90th/50–90th)	4, 3.5,–	hydrocortisone	c.1398+5G>C p.(Arg454Cys)
P3	4.7	Male	46,XY	128.5 (+4.7 SD)	28.0 (+4.5 SD)	13	121/80 (95–99th/95–99th)	5, 8, 4	Hydrocortisone spironolactone GnRHa	p.(Arg141*) P.(Gln73*)
P4	9.3	Male	46,XY	174.4(+6.7 SD)	59.0 (+2.9 SD)	17	150/96 (>99th/>99th)	5, 8, 20	Dexamethasone spironolactone valsartan	P.(Gly267Ser) p.(Gly134Arg)

*Accession no: NM_000497*.

**Table 2:** Biochemical characterization of four patients with 11βOHD.

**Case No**.	**K^**+**^ mmol/L**	**Na^**+**^ mmol/L**	**Cortisol nmol/L**	**ACTH pg/mL**	**T nmol/L**	**P nmol/L**	**LH mIU/mL**	**FSH mIU/mL**	**17-OHP ng/mL**	**ASD ng/mL**	**DHAS μg/dL**	**Renin pg/mL**	**Aldosterone pg/mL**
P1	3.67	137	158.7	242.9	7.59	8.13	<0.1	<0.1	30.5	>10	ND	ND	174.56
P2	4.72	142	80.46	209.2	7.0	7.25	<0.1	1.22	24.73	>10	70.89	ND	ND
P3	4.4	136	22.39	322.7	2.21	2.28	2.6	2.9	14.74	ND	50.35	ND	ND
P4	2.95	143	44.93	545.7	21.13	1.85	4.38	3.64	4.78	6.62	167.5	4.12	93.25
Reference value	3.5–5.5	135–145	171–536	0–46	<0.42	0.15–0.5			0.31–2.3	0.3–3.3(F) 0.6–3.1(M)	0.47–19.4(1–4 y) 2.8–85.2(5–9 y)	4–24	10–160

*ACTH, adrenocorticotropic hormone; T, testosterone; P, progesterone; LH, luteinizing hormone; FSH, follicle-stimulating hormone; 17-OHP, 17-hydroxyprogesterone; ASD, androstenedione; DHAS, dehydroepiandrosterone sulfate; ND, not determined*.

Patients 1 and 2 were raised as boys based on the appearance of the male external genitalia after birth. They were admitted to the local hospital for cryptorchidism at about the age of two. No gonads were palpable in the scrotum or bilateral inguinal canals. Uterus and ovaries were revealed by pelvic ultrasound. Regarding patient 1, laparoscopic exploration of the gonad was performed and tissue biopsy revealed the ovaries at the local hospital. Additionally, physical examination showed high blood pressure (120/80 mmHg, >99th), and external genitalia showed Prader score of 5 (penis 4 cm in length and complete fusion of the labioscrotal folds without hypospadias) while in patient 2 the Prader score was 4 (penis 3.5 cm in length and complete fusion of the labioscrotal folds with hypospadias). Abdominal ultrasound revealed enlargement of bilateral adrenal glands in both patients and hydrocortisone treatment was subsequently started. For patient 1, spironolactone was combined for 3 months, then stopped due to normal blood pressure. In both patients, surgery of feminizing genitoplasty was performed after affirmative diagnosis.

Patient 3 was suspected the diagnosis of CAH with the chief complaint of skin pigmentation and began therapy with glucocorticoid therapy from 3.5 year-old in the local hospital. However, patient compliance with therapy was very poor. He was thereafter admitted to our department for enlargement and erection of penis at the age of 4.7 years. His height was 128.5 cm (+4.7 SD) with advanced bone age (13 years). The following observations were revealed upon physical examination: high blood pressure (121/80 mmHg, 95–99th), skin hyperpigmentation, penile enlargement, and enlarged testicular volume, no pubic hair. Further Gonadotropin-releasing hormone (GnRH) stimulation test showed luteinizing hormone (LH)-peak of 16.9 mIU/mL and follicle-stimulating hormone (FSH)-peak of 6.22 mIU/mL, suggesting that hypothalamic-pituitary-gonadal axis (HPGA) was activated. Abdominal ultrasound revealed enlargement of bilateral adrenal glands. Based on the clinical features and biochemical results, patient 3 was diagnosed with CAH due to 11βOHD, and central precocious puberty was considered. The treatment started with hydrocortisone, spironolactone and GnRH agonist (triptorelin acetate). Patient 4 was admitted to the local hospital for hypokalemic paralysis (Serum K^+^: 2.95 mmol/L, normal range: 3.5–5.5 nmol/L) and hypertension (140/95 mmHg, >99th) at the age of 9 years. Medical imaging showed a mass on the left adrenal gland (5 cm × 2.2 cm) and bilateral adrenal hyperplasia. The patient underwent left adrenal tumor resection. Pathology findings showed adrenocortical oncocytic neoplasm and adrenal cortical hyperplasia. Postoperative clinical examination showed high blood pressure (150/96 mmHg, >99th) that was difficult to control with antihypertensive therapy (nifedipine, valsartan and hydrochlorothiazide). Patient 4 was thereafter admitted to our hospital 3 months after the surgery. Notably, patient 4 showed a tall stature of 174.4 cm (+ 6.7 SD), while his mid-parental height was 173 cm, calculated following the standard procedure from the mean height of the parents (average of the father's and mother's height + 6.5 cm) (13). His puberty advanced to Tanner stage V for pubic hair and testicular volume increased to 20 mL (Tanner IV) with advanced bone age of 17 years. Dexamethasone combined with valsartan and spironolactone treatment was subsequently started, and his blood pressure levels were maintained at the upper limit of normal.

### Mutation Analysis of the *CYP11B1* Gene

**Figure 1:**
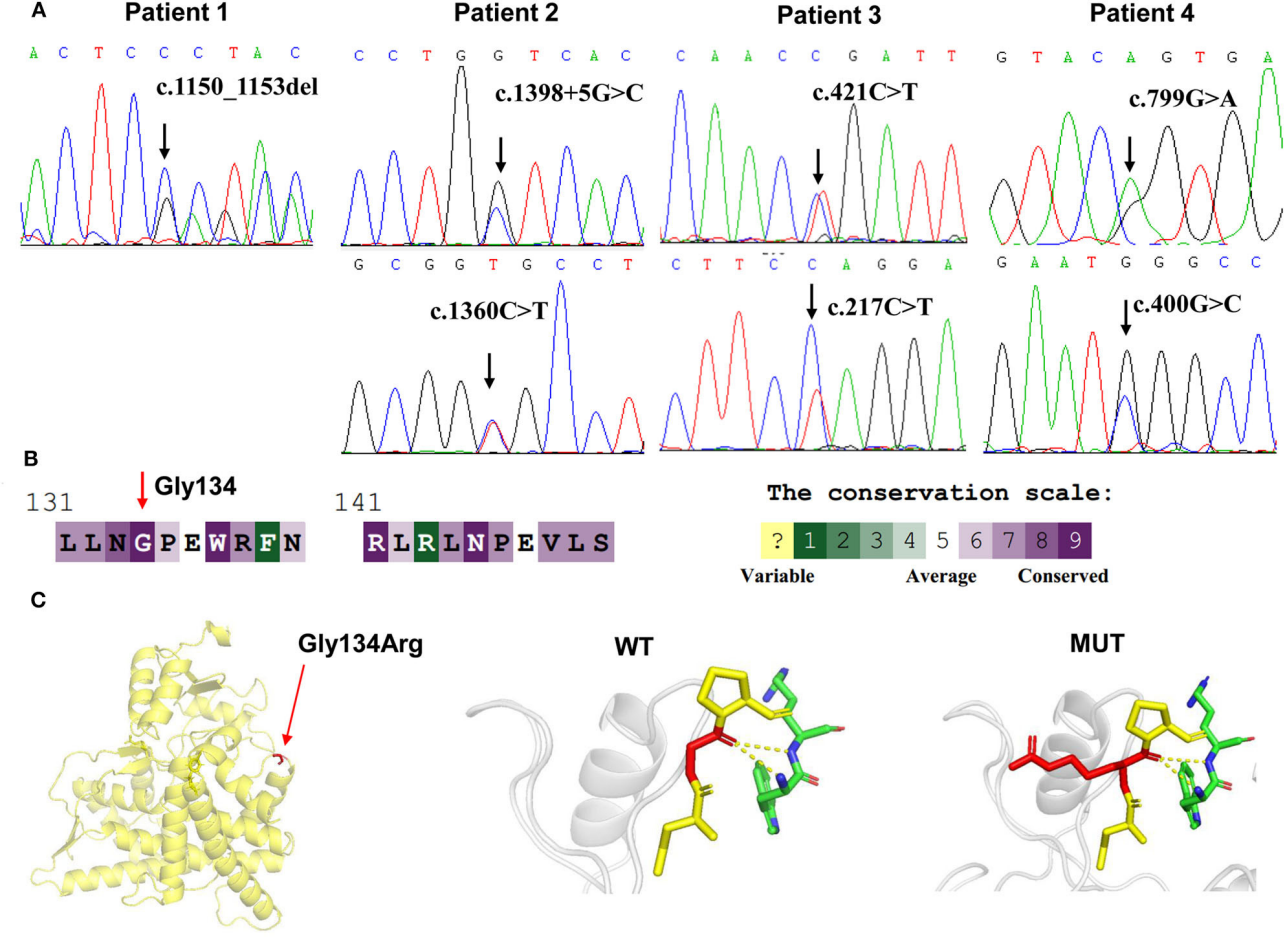
*CYP11B1* gene variants identified in 11βOHD patients. **(A)** Sanger sequencing analysis showing the *CYP11B1* gene mutations in four patients. Arrow indicates the mutation site. **(B)** The evolutionary-conservation scores for residues' (p.Gly134Arg) mutation from different species from the UniRef 90 database. **(C)** Structural visualization of the identified *CYP11B1* missense alteration using X-ray structure of the human *CYP11B1* as a template (PDB accession number: 6M7X). The residues present at our mutation sites are shown as sticks.

**Figure 2:**
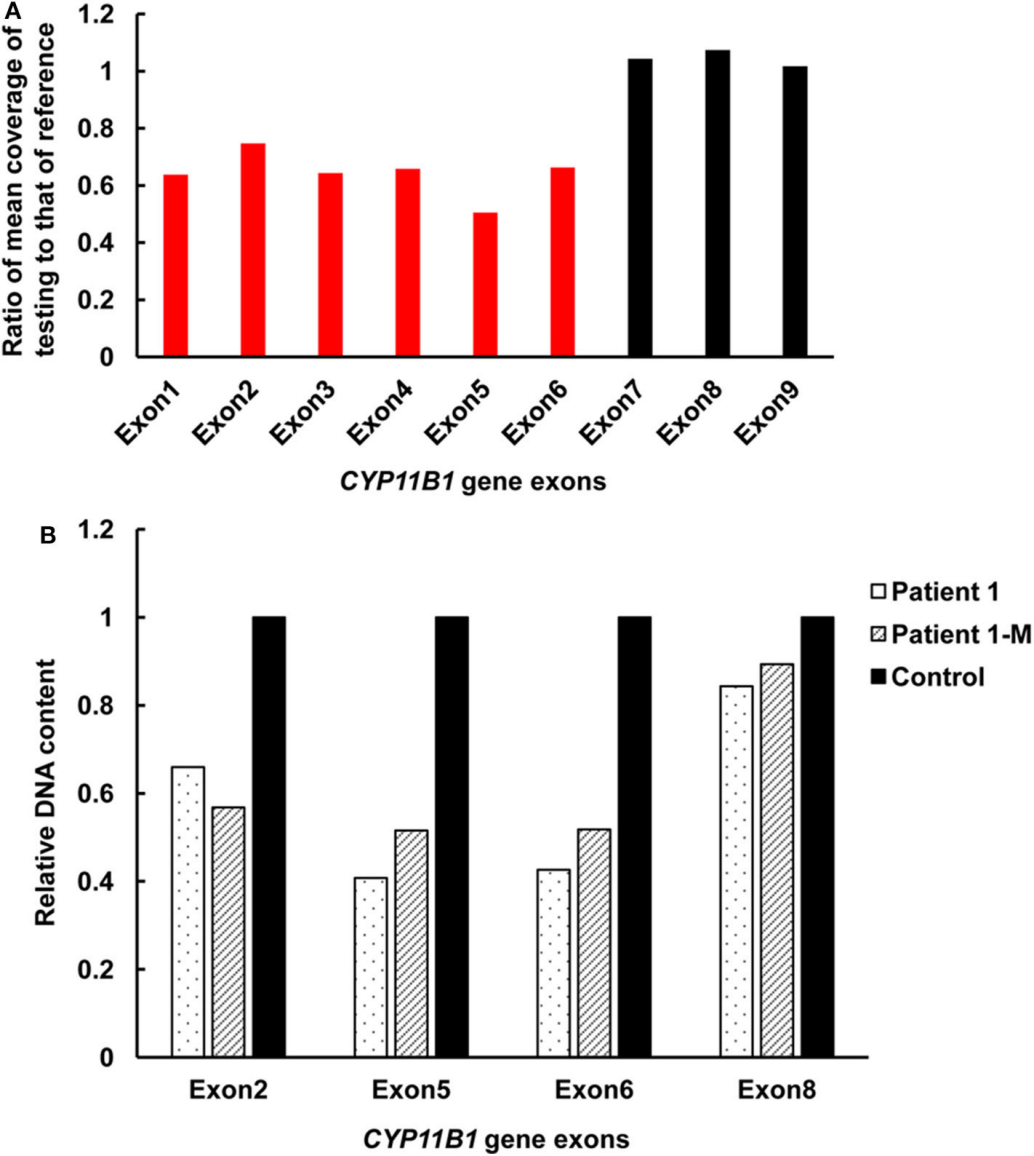
Detection of exonic deletions of the *CYP11B1* gene in patient 1 by CNV analysis using NGS coverage depth and qPCR. **(A)** A heterozygous deletion of exons 1–6 in the *CYP11B1* gene was detected by NGS coverage depth analysis. **(B)** qPCR analysis of relative DNA content in whole blood from patient 1, the mother of patient 1, and the control subject. CNV, copy-number variant; NGS, next-generation sequencing; qPCR, quantitative polymerase chain reaction.

Targeted NGS analysis identified two deletion variants (c.1150_1153del and c.Exons 1–6 del), three missense variants (c.1360C>T, c.799G>A, and c.400G>C), one splicing variant (c.1398+5G>C), and two nonsense variants (c.421C>T and c.217C>T) in the *CYP11B1* (NM_000497) gene in four patients (Table 1). The variants in four patients identified by Sanger sequencing were shown in Figure 1A. Patient 1 had a novel 4-bp CGAG deletion variant c.1150_1153del [p.(Arg384Trpfs^*^45)] in exon 7 from his father and a heterozygous deletion in exons 1 to 6 of the *CYP11B1* gene by NGS coverage depth analysis (Figure 2A). CNV analysis by qPCR revealed the *CYP11B1* gene exons deletion (Exons 1–6 del) in patient 1 inherited from his mother (Figure 2B). Patient 2 carried compound heterozygous for a missense variant c.1360C>T [p.(Arg454Cys)] and a splicing variant c.1398+5G>C in exon 8. Patient 3 had compound heterozygous for a novel nonsense variant c.217C>T [P.(Gln73^*^)] in exon 1 and a recurrent nonsense variant c.421C>T [p.(Arg141^*^)] in exon 3. Patient 4 carried compound heterozygous for a novel missense variant c.400G>C [p.(Gly134Arg)] in exon 3 and a previously reported missense variant c.799G>A [p.(Gly267 Ser)] in exon 4.

### Pathogenicity Analysis of Novel *CYP11B1* Mutations

According to the ACMG-AMP variant interpretation guideline, the novel variants meet the criteria of being likely pathogenic [p.(Arg384Trpfs^*^45) and P.(Gln73^*^)] and uncertain [p.(Gly134Arg)]. The p.(Gly134Arg) was predicted to be potentially damaging by the different bioinformatics software, with a score of 0.00 (damaging) by SIFT, 1.00 (probably damaging) by PolyPhen-2 and−7.84 (deleterious) by Provean. The evolutionary-conservation alignment showed that the 134 residue G is a highly conserved residue (Figure 1B). The three-dimensional molecular model of CYP11B1 showed that Gly134 lies in a loop between B3-and C-helix (Figure 1C).

## Discussion

We identified eight variants of the *CYP11B1* gene in four Chinese patients with 11βOHD, four of which are novel variants. The clinical examination findings of these patients are generally consistent with classical 11βOHD. Notably, serum aldosterone levels in patients 1 and 4 were elevated and normal, respectively, which were expected to be low. We measured aldosterone by chemiluminometric immunoassay (Autobio, China) with a cross reactivity level of <0.1% to other steroid compounds like DOC, 11-deoxycortisol, testosterone and cortisol. Similar to our study, previous reports have shown that elevated aldosterone levels in CAH may be associated with cross-reactivity of high levels of adrenal steroid precursors (14–16), which could explain the aldosterone levels in our patients.

Since salt-wasting is not commonly seen in 11βOHD, the diagnosis of this disorder in these patients was made relatively late. In our study, the diagnosis in all four cases was delayed as well. In addition, about 70% of 46,XX patients with 11βOHD had Prader score 4 or 5 at presentation, so females with 11βOHD are more commonly mis-assigned to the male gender at birth than those with 21OHD (6). Meanwhile, most of males were diagnosed with precocious puberty, hypertension, hyperpigmentation or hypokalemia. Final height was severely impaired in children with 11βOHD, irrespective of age at diagnosis and quality of therapeutic control. The mean adult height was (156.8 ± 7.2) cm in male patients and (153.1 ± 2.9) cm in female patients (10). Bone age was significantly advanced in our cases, yielding a severely compromised predicted final adult height. Unexpectedly, the height of patient 4 wasn't impaired, and he reached his genetical final height, which was not compatible with classic 11βOHD. The underlying mechanism is not clear, though. The three older patients: patient 1, patient 3 and patient 4 presented varying severity of hypertension, which was not the case in the youngest patient (patient 2). This suggests that there is a significant correlation between older age at diagnosis and higher blood pressure. Glucocorticoids can effectively improve blood control, while it might be required to add anti-hypertensive medication in patients with delayed diagnosis of 11βOHD (3). Moreover, some reports have described the role of adrenalectomy in the management of refractory hypertension in 11βOHD (17, 18). Hypokalemia as a result of accumulation of DOC and other steroid precursors is found in a minority of patients with 11βOHD and is not correlated to blood pressure. Similar to the reported patients, hypokalemia in patient 4 resolved with glucocorticoid replacement (19). Adrenal tumor in patient 4 may be caused by chronic ACTH elevation. Long-term stimulation from elevated ACTH levels play a role in the development of adrenocortical tumors in CAH patients. Glucocorticoid treatment can cause the reduction in size of the adrenal masses (20). We support previous recommendation that patients with adrenal masses should be checked for CAH.

The functional severity of *CYP11B1* genotypes may be correlated with age at onset, degree of virilization in female, and age at hypertension. Patient 1, who presented with severe virilization (Prader V) and hypertension at diagnosis before 3 years of age, carried compound heterozygous variants c.[1150_1153del]; [c.Exons1-6del]. The novel variant c.1150_1153del [p.(Arg384Trpfs^*^45)] will probably generate a premature stop codon at the amino acid 429, which will result in the loss of 73 amino acids with severely impaired 11β-hydroxylase enzymatic activity. In close proximity to this variant, c.1179_1180dup [p.(Asn394Argfs^*^37)] was described in a female newborn with severe virilization and a male presenting with hypertension before 2 years of age, respectively (21). The large deletion variant (Exons 3–5del) was reported in a female with complete virilization and raised as male at birth (5). Thus, this larger deletion variant (Exons 1–6del) is predicted to result in completely impaired enzymatic activity.

Patient 2 with compound heterozygous variant c.[1398+5G>C];c.[1360C>T] was diagnosed at 2 years of age with severe virilization (Prader IV) and significantly advanced bone age. Missense variant c.1360C>T [p.(Arg454Cys)] is a hotspot mutation for Han Chinese (22, 23). A Chinese patient was homozygous for this variant, who was raised as male at birth with severe virilization (Prader IV) and diagnosed with hypertension at the age of 16 years (24). The residue Arg454 is localized in close vicinity to Arg453. Therefore, p.(Arg454Cys) may alter heme binding site and result in complete loss of enzyme activity as p.(Arg453Gln) (25). The c.1398+5G>C homozygous mutation was previously described in a 46,XX female with severe virilization of external genitalia at birth (5).

Patient 3 carried compound heterozygous nonsense variants p.(Arg141^*^); p.(Gln73^*^). Vitro expression data showed that p.(Arg141^*^) mutation retained 4.9% of residual 11β-hydroxylase enzymatic activity and almost completely abolished enzymatic activity (22). Thus, the novel variant p.(Gln73^*^) is predicted to result in completely impaired enzymatic activity in the truncated protein, which corresponded to the severe phenotype in patient 3 with hypertension and secondary central precocious puberty at the age of 4.7 years.

Patient 4 harboring compound heterozygous missense variant p.(Gly267Ser); p.(Gly134Arg) presented relatively mild clinical phenotype. Missense variant c.799G>A [p.(Gly267Ser)] activated an alternative splice site within exon 4, leading to a 45-bp deletion in the transcript and a truncated protein (26). The residues His133 and Gly134 are highly conserved and located on a loop between B'-and C-helix. p.Asn133His previously reported in non-classical form of 11βOHD, resulted in ~30–50% residual 11β-hydroxylase activity (27, 28). It seems likely that p.(Gly134Arg) may result in partially disrupted enzyme activity, which is consistent with relatively mild clinical phenotype in patient 4. Taken together, our results support that truncated mutations or mutations that altered the heme binding site in *CYP11B1* gene may account for a more severe phenotype of patients.

## Conclusion

In conclusion, we have found four novel mutations in the *CYP11B1* gene. These results may expand the spectrum of mutations of the *CYP11B1* gene in the Chinese population. Significant heterogeneity of clinical phenotype and low prevalence are largely responsible for the late diagnosis of 11βOHD. Early diagnosis and glucocorticoid treatment are important to prevent complications and improve long-term outcomes.

## Data Availability Statement

The datasets generated for this study can be found in the LOVD database at the following links: https://databases.lovd.nl/shared/individuals/00306720, https://databases.lovd.nl/shared/individuals/00306721, https://databases.lovd.nl/shared/individuals/00306716, https://databases.lovd.nl/shared/individuals/00306718.

## Ethics Statement

The studies involving human participants were reviewed and approved by Children's Hospital of Nanjing Medical University. Written informed consent to participate in this study was provided by the participants' legal guardian/next of kin.

## Author Contributions

QZ, DW, and CW collected samples, performed the experimentation, and analyzed the data. WG and ZJ conceived and designed this study. ZZ and QL collected the clinical samples and clinical data. CW and BZ performed NGS analysis. WG, QZ, and DW wrote the manuscript. All authors have read and approved the final manuscript. All authors contributed to the article and approved the submitted version.

## Conflict of Interest

The authors declare that the research was conducted in the absence of any commercial or financial relationships that could be construed as a potential conflict of interest.

## References

[1] Miller WL. Mechanisms in endocrinology: rare defects in adrenal steroidogenesis. Eur J Endocrinol. (2018) 179:R125–41. 10.1530/EJE-18-0279 29880708

[2] Speiser PW White PC. Congenital adrenal hyperplasia. N Engl J Med. (2003) 349:776–88. 10.1056/NEJMra021561 12930931

[3] White PC. Steroid 11 beta-hydroxylase deficiency and related disorders. Endocrinol Metab Clin North Am. (2001) 30:61–79. 10.1016/S0889-8529(08)70019-7 11344939

[4] Tonetto-Fernandes V Lemos-Marini SH Kuperman H Ribeiro-Neto LM Verreschi IT Kater CE. Serum 21-Deoxycortisol, 17-Hydroxyprogesterone, and 11-deoxycortisol in classic congenital adrenal hyperplasia: clinical and hormonal correlations and identification of patients with 11beta-hydroxylase deficiency among a large group with alleged 21-hydroxylase deficiency. J Clin Endocrinol Metab. (2006) 91:2179–84. 10.1210/jc.2005-1890 16551734

[5] Kandemir N Yilmaz DY Gonc EN Ozon A Alikasifoglu A Dursun A. Novel and prevalent CYP11B1 gene mutations in Turkish patients with 11-beta hydroxylase deficiency. J Steroid Biochem Mol Biol. (2017) 165:57–63. 10.1016/j.jsbmb.2016.03.006 26956189

[6] Khattab A Haider S Kumar A Dhawan S Alam D Romero R. Clinical, genetic, and structural basis of congenital adrenal hyperplasia due to 11beta-hydroxylase deficiency. Proc Natl Acad Sci USA. (2017) 114:E1933–40. 10.1073/pnas.1621082114 28228528 PMC5347606

[7] Mooij CF Parajes S Rose IT Taylor AE Bayraktaroglu T Wass JA. Characterization of the molecular genetic pathology in patients with 11beta-hydroxylase deficiency. Clin Endocrinol. (2015) 83:629–35. 10.1111/cen.12834 26053152

[8] Mimouni M Kaufman H Roitman A Morag C Sadan N. Hypertension in a neonate with 11 beta-hydroxylase deficiency. Eur J Pediatr. (1985) 143:231–3. 10.1007/BF00442149 3872797

[9] Al-Jurayyan NA. Congenital adrenal hyperplasia due to 11 beta-hydroxylase deficiency in Saudi Arabia: clinical and biochemical characteristics. Acta Paediatr. (1995) 84:651–4. 10.1111/j.1651-2227.1995.tb13719.x 7670248

[10] Hochberg Z Schechter J Benderly A Leiberman E Rosler A. Growth and pubertal development in patients with congenital adrenal hyperplasia due to 11-beta-hydroxylase deficiency. Am J Dis Child. (1985) 139:771–6. 10.1097/00006254-198606000-00015 3875277

[11] Bonfig W. Growth and development in children with classic congenital adrenal hyperplasia. Curr Opin Endocrinol Diabetes Obes. (2017) 24:39–42. 10.1097/MED.0000000000000308 27898585

[12] Feng Y Chen D Wang GL Zhang VW Wong LJ. Improved molecular diagnosis by the detection of exonic deletions with target gene capture and deep sequencing. Genet Med. (2015) 17:99–107. 10.1038/gim.2014.80 25032985 PMC4338802

[13] Tanner JM Goldstein H Whitehouse RH. Standards for children's height at ages 2-9 years allowing for heights of parents. Arch Dis Child. (1970) 45:755–62. 10.1136/adc.45.244.755 5491878 PMC1647404

[14] Balcells C Gili T Perez J Corripio R. Pseudohypoaldosteronism without nephropathy masking salt-wasting congenital adrenal hyperplasia genetically confirmed. BMJ Case Rep. (2013) 2013:bcr2012008281. 10.1136/bcr-2012-008281 23370958 PMC3603811

[15] Tuhan HU Catli G Anik A Onay H Dundar B Bober E. Cross-reactivity of adrenal steroids with aldosterone may prevent the accurate diagnosis of congenital adrenal hyperplasia. J Pediatr Endocrinol Metab. (2015) 28:701–4. 10.1515/jpem-2014-0170 25503463

[16] Boddu SK Madhavan S. High aldosterone and cortisol levels in salt wasting congenital adrenal hyperplasia: a clinical conundrum. J Pediatr Endocrinol Metab. (2017) 30:1327–31. 10.1515/jpem-2017-0166 29127765

[17] Chabre O Portrat-Doyen S Chaffanjon P Vivier J Liakos P Labat-Moleur F. Bilateral laparoscopic adrenalectomy for congenital adrenal hyperplasia with severe hypertension, resulting from two novel mutations in splice donor sites of CYP11B1. J Clin Endocrinol Metab. (2000) 85:4060–8. 10.1210/jcem.85.11.6897 11095433

[18] John M Menon SK Shah NS Menon PS. Congenital adrenal hyperplasia 11beta-hydroxylase deficiency: two cases managed with bilateral adrenalectomy. Singapore Med J. (2009) 50:e68–70. 19296015

[19] Goyal A Boro H Khadgawat R. Male gender identity and reversible hypokalemic hypertension in a 46,XX child with 11-Beta-Hydroxylase deficiency congenital adrenal hyperplasia. Cureus. (2019) 11:e5248. 10.7759/cureus.5248 31572633 PMC6760881

[20] Kacem M Said M Achour L Hadj YF Ben KS Mahjoub S. Large bilateral adrenal incidentalomas complicating untreated 11B hydroxylase deficiency in the third decade of life. A case report. Ann Endocrinol. (2000) 61:418–21. 11084392

[21] Bas F Toksoy G Ergun-Longmire B Uyguner ZO Abali ZY Poyrazoglu S. Prevalence, clinical characteristics and long-term outcomes of classical 11 beta-hydroxylase deficiency (11BOHD) in Turkish population and novel mutations in CYP11B1 gene. J Steroid Biochem Mol Biol. (2018) 181:88–97. 10.1016/j.jsbmb.2018.04.001 29626607

[22] Zhang M Liu Y Sun S Zhang H Wang W Ning G. A prevalent and three novel mutations in CYP11B1 gene identified in Chinese patients with 11-beta hydroxylase deficiency. J Steroid Biochem Mol Biol. (2013) 133:25–9. 10.1016/j.jsbmb.2012.08.011 22964742

[23] Wang X Nie M Lu L Tong A Chen S Lu Z. Identification of seven novel CYP11B1 gene mutations in Chinese patients with 11beta-hydroxylase deficiency. Steroids. (2015) 100:11–6. 10.1016/j.steroids.2015.04.003 25911436

[24] Sun SY Zhang MN Yang J Zhang HJ Liu JM Hong J. [Clinical and genetic analysis of 11beta-hydroxylase deficiency]. Zhonghua Yi Xue Za Zhi. (2011) 91:2999–3002. 22333028

[25] Krone N Grotzinger J Holterhus PM Sippell WG Schwarz HP Riepe FG. Congenital adrenal hyperplasia due to 11-hydroxylase deficiency–insights from two novel CYP11B1 mutations (p.M92X, p.R453Q). Horm Res. (2009) 72:281–6. 10.1159/000245930 19844114

[26] Soardi FC Penachioni JY Justo GZ Bachega TA Inacio M Mendonça BB. Novel mutations in CYP11B1 gene leading to 11 beta-hydroxylase deficiency in Brazilian patients. J Clin Endocrinol Metab. (2009) 94:3481–5. 10.1210/jc.2008-2521 19567537

[27] Joehrer K Geley S Strasser-Wozak EM Azziz R Wollmann HA Schmitt K. CYP11B1 mutations causing non-classic adrenal hyperplasia due to 11 beta-hydroxylase deficiency. Hum Mol Genet. (1997) 6:1829–34. 10.1093/hmg/6.11.1829 9302260

[28] Barr M MacKenzie SM Wilkinson DM Holloway CD Friel EC Miller S. Functional effects of genetic variants in the 11beta-hydroxylase (CYP11B1) gene. Clin Endocrinol. (2006) 65:816–25. 10.1111/j.1365-2265.2006.02673.x 17121536

