# Supplementary material for: Clinical and Molecular Analysis of Four Patients With 11β-Hydroxylase Deficiency
Source: Front Pediatr. 2020 Jul 24;8:410. doi: 10.3389/fped.2020.00410 (PMC7396487; doi:10.3389/fped.2020.00410)
Supplement: Supplementary file 1 [file Table_1.DOCX]

[**Supplementary Table 1**](https://www.frontiersin.org/articles/10.3389/fgene.2020.00081/full#h13) The primer pair sequences for qPCR

| Exons | Sequence |
| --- | --- |
| CYP11B1-E2-Forward | 5’-GTACGACTTGGGAGGAGCAG-3’ |
| CYP11B1-E2-Reverse | 5’-CAGCAAGAACACGCCACATT-3’ |
| CYP11B1-E5-Forward | 5’-CGGATAGGCGACAACTGTAT-3’ |
| CYP11B1-E5-Reverse | 5’-ATGGCATCTGGCGACAGT-3’ |
| CYP11B1-E6-Forward | 5’-CTGTCGTTCTCAGGGTATGC-3’ |
| CYP11B1-E6-Reverse | 5’-AAGAGCGTCATCAGCAAGG-3’ |
| CYP11B1-E8-Forward | 5’-ATTGGTGCGCGTGTTCCT-3’ |
| CYP11B1-E8-Reverse | 5’-CGTGGTAGAAGTTCCTGCC-3’ |
